# A Mobile App Development Guideline for Hospital Settings: Maximizing the Use of and Minimizing the Security Risks of "Bring Your Own Devices" Policies

**DOI:** 10.2196/mhealth.4424

**Published:** 2016-05-11

**Authors:** Soleh U Al Ayubi, Alexandra Pelletier, Gajen Sunthara, Nitin Gujral, Vandna Mittal, Fabienne C Bourgeois

**Affiliations:** ^1^ Innovation & Digital Health Accelerator Boston Children's Hospital Boston, MA United States; ^2^ United States Digital Service The White House Washington, DC United States; ^3^ General Pediatrics Boston Children's Hospital Boston, MA United States

**Keywords:** BYOD, guideline, safeguard, custom application, hospital settings, security, privacy, mobile application, electronic medical records

## Abstract

**Background:**

Hospitals today are introducing new mobile apps to improve patient care and workflow processes. Mobile device adoption by hospitals fits with present day technology behavior; however, requires a deeper look into hospital device policies and the impact on patients, staff, and technology development. Should hospitals spend thousands to millions of dollars to equip all personnel with a mobile device that is only used in a hospital environment? Allowing health care professionals to use personal mobile devices at work, known as bring-your-own-device (BYOD), has the potential to support both the hospital and its employees to deliver effective and efficient care.

**Objective:**

The objectives of this research were to create a mobile app development guideline for a BYOD hospital environment, apply the guideline to the development of an in-house mobile app called TaskList, pilot the TaskList app within Boston Children’s Hospital (BCH), and refine the guideline based on the app pilot. TaskList is an Apple operating system (iOS)-based app designed for medical residents to monitor, create, capture, and share daily collaborative tasks associated with patients.

**Methods:**

To create the BYOD guidelines, we developed TaskList that required the use of mobile devices among medical resident. The TaskList app was designed in four phases: (1) mobile app guideline development, (2) requirements gathering and developing of TaskList fitting the guideline, (3) deployment of TaskList using BYOD with end-users, and (4) refinement of the guideline based on the TaskList pilot. Phase 1 included understanding the existing hospital BYOD policies and conducting Web searches to find best practices in software development for a BYOD environment. Phase 1 also included gathering subject matter input from the Information Services Department (ISD) at BCH. Phase 2 involved the collaboration between the Innovation Acceleration Program at BCH, the ISD Department and the TaskList Clinical team in understanding what features should be built into the app. Phase 3 involved deployment of TaskList on a clinical floor at BCH. Lastly, Phase 4 gathered the lessons learned from the pilot to refine the guideline.

**Results:**

Fourteen practical recommendations were identified to create the BCH Mobile Application Development Guideline to safeguard custom applications in hospital BYOD settings. The recommendations were grouped into four categories: (1) authentication and authorization, (2) data management, (3) safeguarding app environment, and (4) remote enforcement. Following the guideline, the TaskList app was developed and then was piloted with an inpatient ward team.

**Conclusions:**

The Mobile Application Development guideline was created and used in the development of TaskList. The guideline is intended for use by developers when addressing integration with hospital information systems, deploying apps in BYOD health care settings, and meeting compliance standards, such as Health Insurance Portability and Accountability Act (HIPAA) regulations.

## Introduction

Smartphones help individuals perform many functions and are now considered a critical tool in some workplaces. In the United States, smartphone market penetration reached 74% to 77% in the 3rd quarter of 2015 [1,2]. In health care, the market penetration is higher; smartphones were adopted by 96% of physicians [3]. Health care professionals are relying more on their smartphones to access medical information, clinical tools, or patient information [4-10]. According to a recent study [11], 89% of health care workers use their smartphone for work purposes, and another survey [3] found that 96% of physicians interviewed used smartphones as their primary device to support clinical communications. Of the 130 hospitals in the United States, 85% (111/130) support the use of personal devices, including smartphones, at work [12]. The ability for professionals to use their personal mobile devices at work is widely known as bring-your-own-device (BYOD).

As BYOD becomes more popular across industries, hospitals are also beginning to adopt BYOD policies for health care staff. The explosive adoption of mobile device usage by health care professionals [3,13] has led to the growth of new patient care mobile apps that are having positive impact on patient care [4-8,10,14-17]. Implementing BYOD eliminates the need for organizations to purchase mobile devices and saves money in the long run. By embracing a more mobile workplace, health care organizations can support the work demand of staff that works across multiple clinics and hospitals. However the adoption of BYOD increases in hospital settings, challenges still exist in the areas of privacy-security compliance and information technology (IT) management.

A number of privacy-security compliance risks arise when applying BYOD to health care environments. For instance, if a user is interacting with their mobile device in a hospital to retrieve protected health information (PHI), security risks may emerge if sensitive data is exposed. As a result, BYOD has created security concerns for many hospitals. A survey revealed 53% of health care professionals used smartphones or tablets for work purposes through unsecured WiFi networks; 41% of the devices were not password protected; and only 52% reported having the Bluetooth discoverable mode disabled on their smartphones [11]. As BYOD becomes more of an established policy at hospitals, hospital IT departments will need to manage a large variety of mobile devices that require hospitals to increase technical support resources in already resource- constrained hospital IT departments.

Nevertheless, BYOD is inevitable; health care organizations should focus on how to enable effective and efficient BYOD policies rather than to restrict the use of personal mobile devices. Several approaches exist to limit security risk and create an acceptable use of BYOD in health care settings. A few highlighted methods include: (1) incorporating BYOD policy text within employment agreements, (2) developing mobile device management (MDM) procedures, and (3) developing guidelines for how mobile apps should be developed to minimize security risks. First, health care organizations need to develop clear guidance for employees on how to use personal mobile devices for work (ie, texting, pictures). At health care organizations, incorporating these clear policies within employment agreements will help stress the importance of maintaining care and confidentiality when handling PHI. Second, leveraging MDM technology helps secure, monitor, manage and support mobile devices across enterprises. MDM functionalities typically include over-the-air (OTA) distribution of apps, data and configuration settings, and security settings for mobile devices. The functionalities were designed with the intent to minimize operational costs, system downtime, and business risks [18]. Third, a framework for hospital employees developing mobile apps will help address the needs to design and develop the mobile app to protect PHI.

The first two approaches have been developed and widely implemented [12,19]. Whereas the first two approaches do not cover the issues of custom mobile apps complying with BYOD concerns and policies, the third approach provides the opportunity to focus on identifying technological development methods that fit within the concerns and policies. Unfortunately, unlike the first two approaches, the technological development approach is still a domain that is being defined. Therefore, this paper is written with the purpose to fill the gap by proposing a guideline that can be used by app developers, designers, and product managers to develop apps complying with BYOD and associated hospital security risks. This paper offers a descriptive guideline on how mobile apps can be designed for hospital BYOD environments while maintaining their existing security policies per Health Insurance Portability and Accountability Act (HIPAA) regulations. The proposed guideline applies only to tablets, mini tablets, and smartphone devices. Due to the maturity of security measures already established and the ease of authentication requirements and usability compared with other mobile devices, laptops will not be included in this paper. To focus the scope of the guideline, other handled devices will also not be included.

## Methods

### Development

Multiple steps were taken to develop the Boston Children’s Hospital’s (BCH) BYOD mobile application development guideline. The steps include understanding the current standards and taking into consideration established policies within hospital settings through Internet research. The research established the theoretical and practical foundation of how to go about creating apps within a BYOD environment. The study led to identifying potential BYOD risks when accessing patient information, understanding how other organizations developed their BYOD guidelines and risks associated with them, and developing potential solutions and recommendations for a hospital-appropriate mobile app development guideline for BYOD. After conducting this research, discussions were conducted with both external and internal security professionals and the team interviewed Information Services Department (ISD) leaders at BCH. Finally, we developed a hospital mobile app development guideline and named it BCH BYOD Mobile Application Development Guideline.

### Implementation

After establishing the guideline, the team developed a mobile app, called TaskList, which could adhere to the privacy and security concerns related to BYOD in health care settings. TaskList is an Apple operating system (iOS)-based app designed for medical residents at BCH to monitor, create, capture, and share daily collaborative tasks associated with patients [20]. The TaskList app is integrated with the electronic medical record (EMR), Cerner and EPIC systems, BCH email system, and BCH lightweight directory access protocol (LDAP) authentication system. Cerner and EPIC systems offer an integrated suite of software that support functions related to patient care and hospital operation, such as patient registration and scheduling, clinical systems for providers, administrative systems for pharmacists, and billing systems for insurers. BCH is a leading pediatric hospital serving as one of the largest pediatric medical centers in the United States [21]. It offers a full range of health care services for infants, children, and adolescents [21]. The hospital has over 5500 mobile devices connected to its network, the majority of which are employee-owned iPhones and iPads. Due to the popularity of BYOD at BCH, TaskList proved to be an appropriate test case to determine app requirements within a BYOD environment and to test the BCH BYOD Mobile Application Development Guideline.

## Results

### BCH BYOD Mobile Application Development Guideline

From our research and subject matter interviews externally and internally to BCH, we created 14 practical recommendations for the BCH BYOD guideline. Table 1 describes each recommendation and how it relates to developing a mobile app.

**Table 1:** Summary of BCH BYOD guideline to safeguard custom application in hospital settings.

No.	Risks	Guidelines and Recommendations
1	Unauthorized access to app and decreased productivity	Adopt enterprise-standards but usable authentication
		Implement RBAC^a^
2	Unauthorized access to data	Implement at least three layers of security on data transmission (transport layer security, access control, and content security)
		Allow apps to work on internal networks or VPN^b^ only
3	Data transmission to unauthorized parties	Protect the mobile app’s notifications
4	Unauthorized access to apps and data	Prevent apps from working on jail-broken devices
		Allow apps to only work on encrypted-devices or devices with pass-codes
5	Unauthorized access to data	Require apps to use minimal cache
6	Unauthorized access to the app	Enforce automatic logoff
7	Data transmission to unauthorized parties	Limit copy data and print screen functionalities
		Limit backup on Cloud services
8	App distribution to unauthorized parties	Distributing the app: Implement internal over-the-air installation and app updates
9	Unauthorized access to app	Implement remote wipe out functionality
		Implement ability to disconnect and block a user anytime

^a^role-based access control.

^b^virtual private networks.

#### Authentication and Authorization

##### Adopt Enterprise-Standards With Convenient Authentication

User verification is a crucial component of secured systems, especially for medical-related systems. The verification provides access to valuable information and offers personalized services. Most health care systems require individual and enterprise standard authentication with the ability to time-out a user after a period of inactivity. The enterprise authentication procedure at times requires at least three combinations of keyboards (alphabet, numbers, and special characters) that can be cumbersome to switch between when using a mobile device. Because productivity is impaired by these hassles, this barrier should be minimized for clinicians. Designing an authentication process that complies with the required security standards, while still being usable and convenient, should be built into the app.

##### Implement Role-Based Access Control

Within an organization,  are created for various job functions. The permissions and security measures to perform certain operations and access specific features within an app should be assigned based on roles. Employees are assigned particular roles, and through role assignments acquire computer permissions to perform particular computer-system functions. This is widely recognized as role-based access control (RBAC) and has been endorsed by the US government [22,23]. RBAC simplifies security management by providing a role hierarchy structure that eventually reduces a business risk caused by complex user management.

#### Data Management

##### Implement at Least Three Layers of Security on Data Transmission (Transport Layer Security, Access Control, and Content Security).

Following a recommendation from the US National Institute of Standards and Technology [22], at least three layers of security measures of data transaction need to be implemented for secure data transmission. This includes using Secure Sockets Layer (SSL) as a data transfer protocol, ensuring timely restricted and authenticated transactions (session-based access as access control), and making the data transferred in the channel securely encrypted (content security). Implementing the three levels of protection layers is one of the simplest-and most important-security measures to reduce the risk of data being accessed and used by unauthorized parties.

##### Allow Apps to Work on Internal Networks or Virtual Private Networks Only

A virtual private network (VPN) is a group of computing devices (computer, tablet, printer, and mobile phone) networked together over a public network, namely, the Internet. VPN allows devices to connect to remote resources when they are not physically on the same local area network (LAN). VPN enables mobile employees, telecommuters, business partners, and others to take advantage of locally available, high-speed broadband to gain access to the enterprise’s network. VPN provides a high level of security, using advanced encryption and authentication protocols to safeguard data from snoops, data thieves, and other unauthorized parties. Limiting the apps to work on internal networks or VPN-only networks assures one simple security practice that prevents unauthorized parties to snoop during data transmission.

##### Protect the Mobile App’s Notifications Appropriately

A good practice is to always secure PHI and to limit sending non-PHI to third parties outside of a hospital network. On mobile devices (tablets or smartphones), an app is designed to be inactive while it is running in the background due to its limited resources. When someone sends a message to an app idling in the background there is no way to deliver that message other than using a push notification feature from its operation system. Examples of notification features include the Apple Push Notification System (APNS) for iOS devices or Google Cloud Messaging (GCM) for Android devices that display limited text on a mobile device’s home screen to alert a user that a message is available on the app. In this case, the best practice is to send simple non-PHI content via notifications, for example: “You have a new update.” Then, when the user responds to the notification, the app will pull the associated PHI from internal hospital resources and display the PHI to the users. This practice will allow an app to push a notification/message to users without having to breach HIPAA rules by not sending the associated PHI to the third parties.

#### Safeguarding the App Environment

##### Prevent Apps From Working on Jail-Broken Devices

Jail breaking is a process used to modify the operating system running on a device. The process includes removing standard-imposed security and restrictions, allowing unsecured or illegal operations, such as installing malicious code or data sniffing code. The jail-breaking process may also cause a device to function incorrectly or stop working. Therefore, including a requirement that health care apps should not operate or function on jail broken devices is mandatory.

##### Allow Apps to Only Work on Encrypted-Devices or Devices With Pass-Codes

Ideally, in the case where protected medical data has been accessed by unauthorized parties, the data still has one more layer of protection: encryption. The parties will not understand the encrypted data without a proper key to open it. When protected medical data is stolen and not encrypted, the general attorney’s office may get involved. Under certain circumstances, data breaches of unencrypted protected medical data are required to be reported to the general attorney’s office. In 2009, the US Government enacted the Health Information Technology for Clinical Health Act (HITECH) that requires health care organizations to notify patients if their health records have been compromised. Therefore, preventing the apps from being installed on unencrypted devices is of paramount importance.

##### Require Apps to Use Minimal Cache

A cache is a temporal repository for stored data that is used to expedite the process of retrieving data from remote storage. Retrieving data can be quicker because an app will check the cache for previously stored information without having to recompute or refetch the data from its original remote locations (eg, database server). While there are many reasons for using cache in app design, the security threat caching presents is high when handling PHI. Caching increases the risk of unauthorized parties being able to access stored sensitive information. When designing a mobile app within a BYOD environment, it is recommended to use cache in a limited capacity while ensuring quick app performance.

##### Enforce Automatic Logoff

An automatic logoff functionality will terminate an app when there is no activity on the device, such as screen touch and keyboard activity, after a predefined amount of time. This policy will protect access to the app when the device is intentionally or unintentionally left unattended while the app is open. Another valid security concern results from users leaving their accounts unattended for lengthy periods of time. This situation allows an intruder to take control of the user’s terminal, potentially compromising the security of the system. Therefore, defining automatic log off for both the app and account access is an important consideration to ensure accurate authentication and access.

##### Limit Copy Data and Print Screen Functionalities

In general environment settings, once the information is displayed on a screen, there is no way to prevent users from spreading that information. Nevertheless, there is one way to reduce the risk of data being spread uncontrollably: preventing a user to print the screen. Print-screen is a feature on a mobile device that allows anyone to copy their screen and save it on their device to send to other people. Therefore, designing apps to detect such activity should be required in order to limit the improper dissemination of protected health information.

##### Limit Backup in Cloud Services

As a standard mechanism for backup data, most mobile devices have an optional cloud-based storage for its apps. Cloud data centers are located all over the world, with a typical customer having limited knowledge as to where the data is actually stored. HIPAA mandates that health care organizations have absolute control of sensitive information. Thus, a feature detecting and preventing mobile devices from storing or backing up sensitive information to its cloud storage is necessary for any clinical app running on a BYOD device unless the organization and their Cloud service providers sign a HIPAA Business Associate Agreement (BAA). A HIPAA BAA is a contract between a HIPAA covered entity such as a hospital and a HIPAA business associate (BA), such as a third party contractor, with the main purpose of protecting PHI in accordance with HIPAA guidelines [24]. This agreement has been a requirement since January 25, 2013 when the US Department of Health and Human Services released the Omnibus Rule, which finalized all the former interim rules for HIPAA and HITECH compliance including that of Cloud data services.

#### Remote Enforcement

##### Distributing the App: Implement Internal Over-the-Air Installation and App Updates

General apps for mobile devices are required to be distributed to users through an app market (eg, Apple App Store, Google Play, Windows Phone Store, etc.). However, in many cases, health care apps are designed to be downloaded and installed by hospital employees only and not the public. One solution to the distribution challenge is to attach the device to a development computer, add the device as a development tool, and install the app manually. As the number of users grow, the solution will be tedious and not an efficient distribution process. Therefore, implementing an easy, in-hospital, OTA installation and update process will reduce the burden both on users and IT personnel. Eliminating manual installation lowers the security risk of distributing the mobile app to unauthorized users.

##### Implement Remote Wipe Out Functionality

Nearly 1.6 million smartphones are stolen annually in the United States [25] and theft of these devices is the number one reason that the integrity of information is compromised [26]. Remote wipe is a security feature that allows an IT administrator or device owner to send a command to a device to delete a device’s data. What remote wipe accomplishes can depend on the device, its specific operating system, and any third-party MDM software installed on the device. In an app context, there is one feature called local or auto wipe that clears a mobile device after a prespecified number of failed login attempts, moves outside of a defined physical boundary (geo-fencing), or any other scenarios. Many of the current MDM technologies allows remote wipe of all the data on a device. In some cases, a hospital has the right to wipe out hospital-related data only (a selective folder named sandbox) and not personal data. Thus, designing an app that has the capability to remote wipe a selected folder/data file is of paramount importance, especially when MDM technology is not deployed in the organization.

##### Implement Ability to Disconnect and Block a User Anytime

Under HIPAA regulations, organizations are required to know which users are accessing PHI and manage their access appropriately. As clinical residents and others change employment status or leave a hospital organization, the need to update each person’s role requires the need to update app credentials. This feature may not be available in a current user management system such as active directory. Active directory is a Windows Server feature that allows network administrators to authenticate and manage users. Therefore, implementing a user management dashboard for app administrators to manage (add, disconnect, and delete) users is strongly suggested.

### TaskList BYOD Features Based on the Guideline

After the initial research and interviews with IT experts internal to BCH, we applied the BCH BYOD guideline to the TaskList design and development. TaskList incorporated 12 of 15 recommendations.

#### Authentication and Authorization

##### Adopt Enterprise-Standards but Convenient Authentication

**Figure 1:**
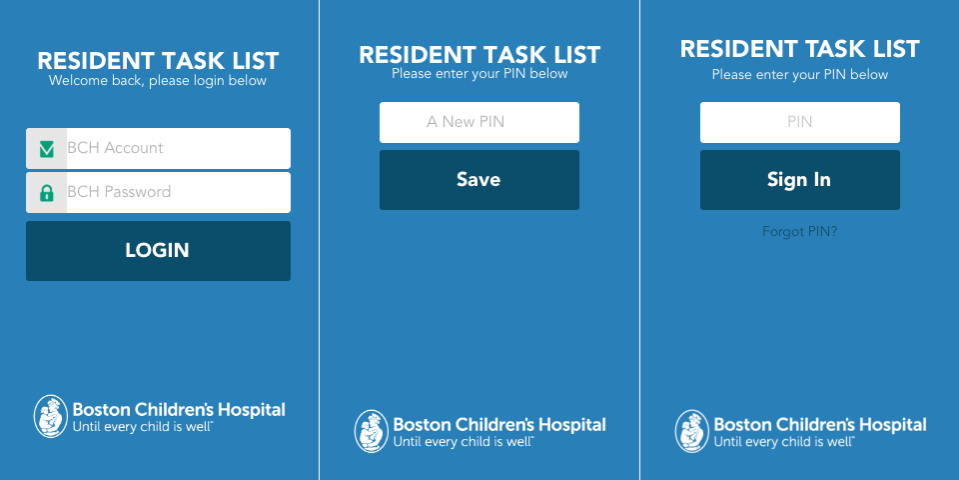
TaskList enterprise authentication and PIN.

In TaskList, to develop an authentication with enterprise standards, while still being easily accessible and convenient, we combined BCH enterprise authentication with a personal identification number (PIN). The app requires users to use enterprise authentication to login for the first time. This credential is valid for 24 hours (Figure 1, left) and must be changed the following day. After a user successfully logs in to TaskList using an enterprise account, the app will ask the user to create a four-digit PIN (Figure 1, middle). If successful, the PIN is valid for 24 hours (Figure 1, middle). Therefore, whenever a user opens TaskList after being logged off (30 minutes of inactivity logs off a user) or after the device is locked, the user would only need to enter the four-digit PIN (Figure 1, right). ISO 9564-1, the international standard for PIN management and security, allows for PINs to between four and 12 digits [27]; but for usability reasons, TaskList uses only four digits which is the most commonly used PIN length [28].

##### Implement Role-Based Access Control

This feature has not been implemented in the TaskList pilot because the users all had the same role. If TaskList should expand beyond the current pilot, role-based access management will be implemented.

#### Data Communication

##### Implement at Least Three Layers Of Security on Data Transmission (Transport Layer Security, Access Control, and Content Security)

In TaskList, we implemented Web-service data communication, store and retrieve, which can be accessed through SSL only. This guideline is also part of the BCH data communication standards. In addition, every Web-service call requires a valid session from BCH enterprise accounts as one of the parameters. This allows the app server to check whether the call should be processed or ignored. Only PHI-related information was encrypted to meet encryption guidelines and to balance the resources to encrypt or decrypt.

##### Allow Apps to Only Work on Internal Networks or Virtual Private Networks

**Figure 2:**
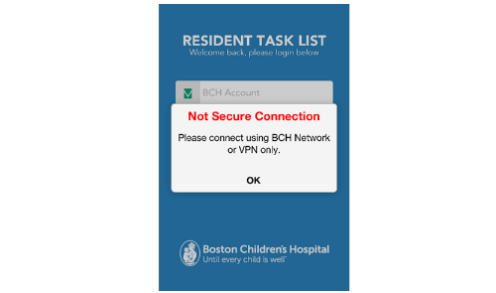
Tasklist runs on secured network only.

TaskList is able to detect whether it is launched on a BCH internal network (WiFi) or VPN. When the app detects that the access comes from an unsecured network (not internal or VPN), a message pops up alerting users that the app cannot be accessed (Figure 2).

##### Protect the Mobile App’s Notifications

**Figure 3:**
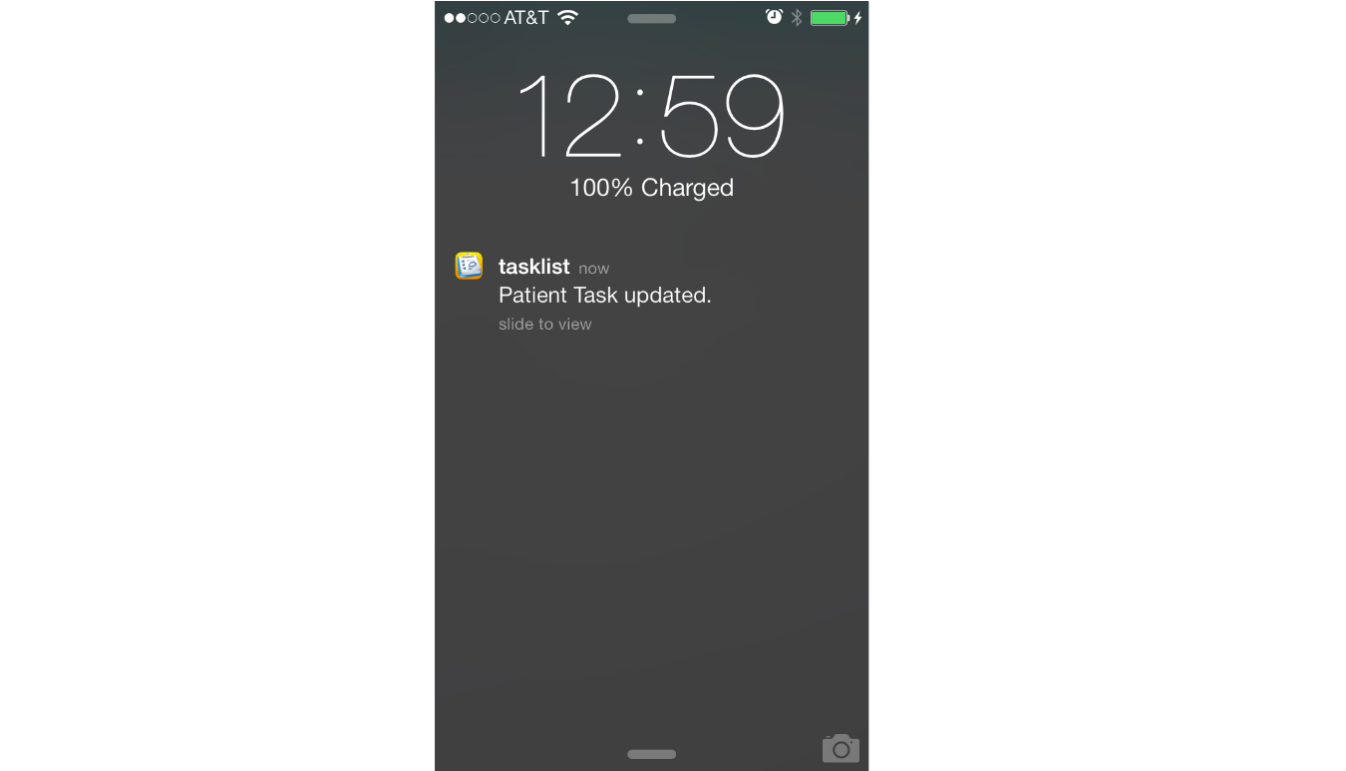
Tasklist limited notifications.

The only external-BCH system that TaskList connects to is the APNS. TaskList may not always be active on a mobile device resulting in the loss of connectivity between the app client and server. When the server sends a notification to the TaskList app in its dormant state, typically APNS is used. To comply with HIPAA, TaskList sends simple non-PHI content via the notifications, such as: “You got a new update” (Figure 3). When the user clicks the notification, TaskList will open from the background and show the information that triggered the notification. This allows the user access to PHI from the hospital network.

#### System-User Interaction/Local Environment

##### Prevent Apps From Working on Jail-Broken Devices

**Figure 4:**
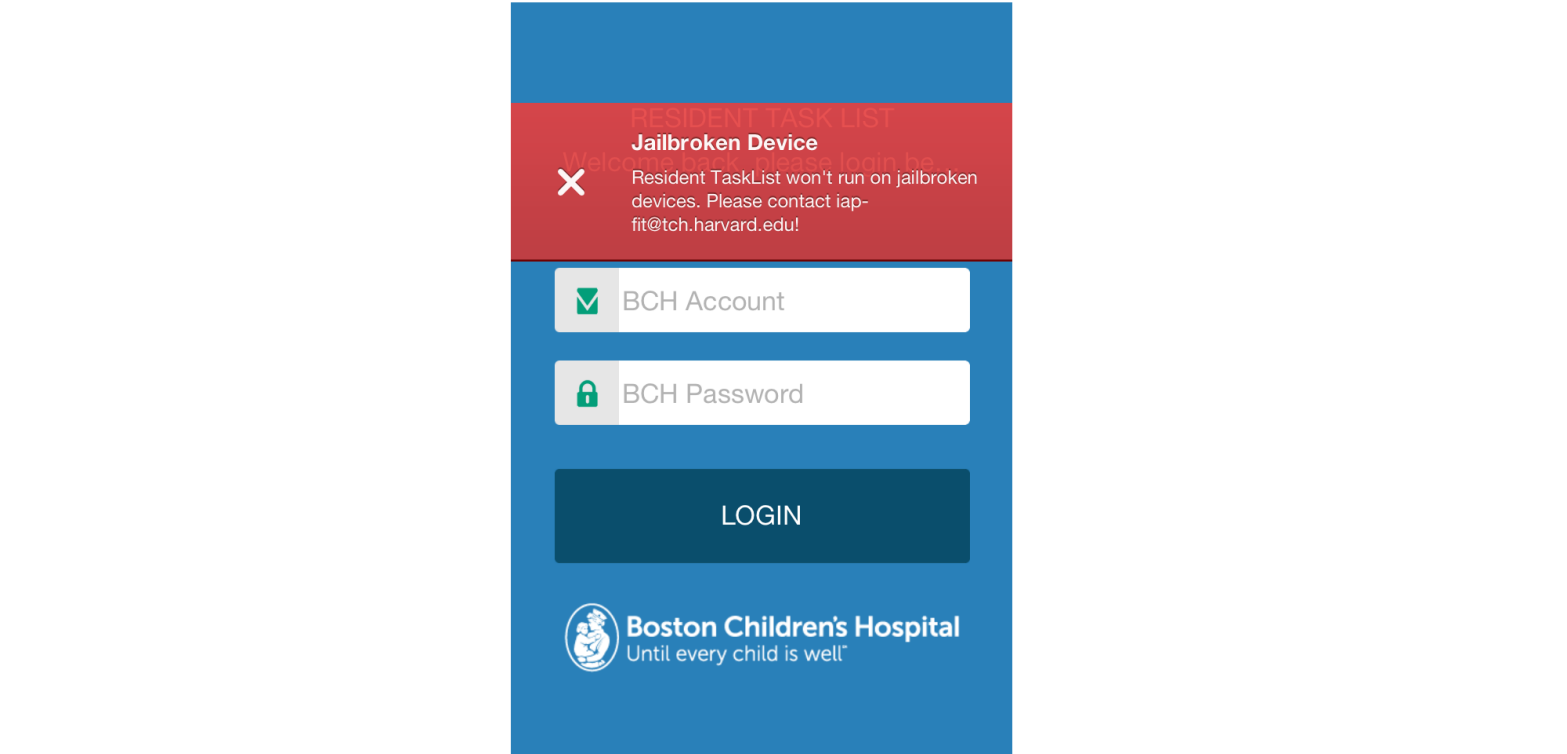
TaskList does not run on jail-broken devices.

TaskList is able to detect whether it is operating on jail-broken devices. When the app detects that the access comes from unsecured devices (jail-broken), a message will pop up and notify users that the app cannot be accessed from the device (Figure 4).

##### Allow Apps to Only Work on Encrypted-Devices or Devices With Pass-Codes

BCH requires that all laptops and mobile devices connected to BCH network or that are used for BCH-related work must have encryption software installed to protect against potential breaches. Meanwhile, Apple provides a dedicated advanced encryption standard (AES) 256-bit hardware encryption for all data stored on iOS devices [29]. Some iOS apps are designed not to be functional on a device without a passcode and have the ability to display a message saying that this app only runs on passcode protected devices. For the TaskList project, passcode detection and restriction were not implemented because we required the app to only function when connected to the BCH network profile. The profile forces all mobile devices to have a passcode to access the BCH internal network and VPN.

##### Enforce Apps to Work With Minimal Cache

Caching leads to security issues while at the same time providing convenience in improving access speed to end users. In the TaskList app, three levels of cache were designed. Level 1: iOS standard cache means that the app allows an iOS to manage its cache; Level 2: on-memory only cache allows the cache to be stored on the device’s memory (not on the hard disk), whenever the memory is full or the app is closed, the cache will be erased; Level 3: no-cache implies that every time data is needed, it will be pulled from the original source. On a standard operation, when the delay to pull and process data is generally accepted by users (less than 15 seconds to populate rarely accessed data such as patient lists and less than 1 second to populate other data), then the ability to have no cache is balanced with performance expectations. The TaskList app is setup to use no cache (Level 3).

##### Enforcing Automatic Logoff

**Figure 5:**
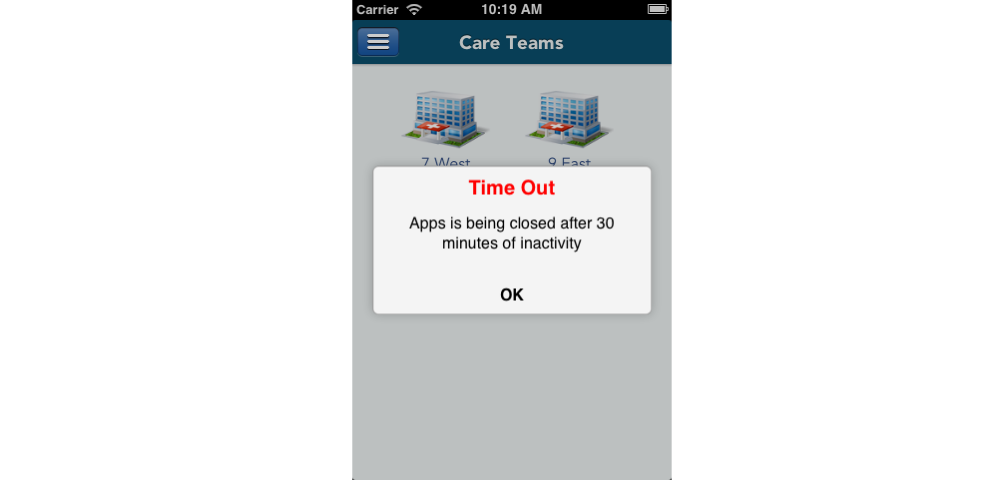
TaskList will be closed after 30-minute of inactivity.

In the TaskList app, the automatic logoff feature will terminate the app when there is no touch on a device screen after 30 minutes (Figure 5). If it is also within the 24-hour authentication window, a new PIN login will be prompted for the user.

##### Limit Copy Data and Print Screen Functionalities

In the TaskList project, when users are detected using the print screen operation (clicking the home and device power buttons together) a message is displayed informing the user that print screen cannot be done. This will not prevent, but does limit the unauthorized spread of information.

##### Limit Backup on Cloud services

As TaskList does not store any data locally on devices and uses no-cache, the feature that prevents storing/backup data on the Cloud was not implemented. In the future, when many departments join the pilot, the data get bigger and more time is needed to load the data from server. Consequently, storing some of the data locally on devices and using on-memory cache and preventing backup the data to Cloud services are required.

#### Remote Enforcement

##### Distributing the App: Implement Internal Over-the-Air Installation and App Updates

**Figure 6:**
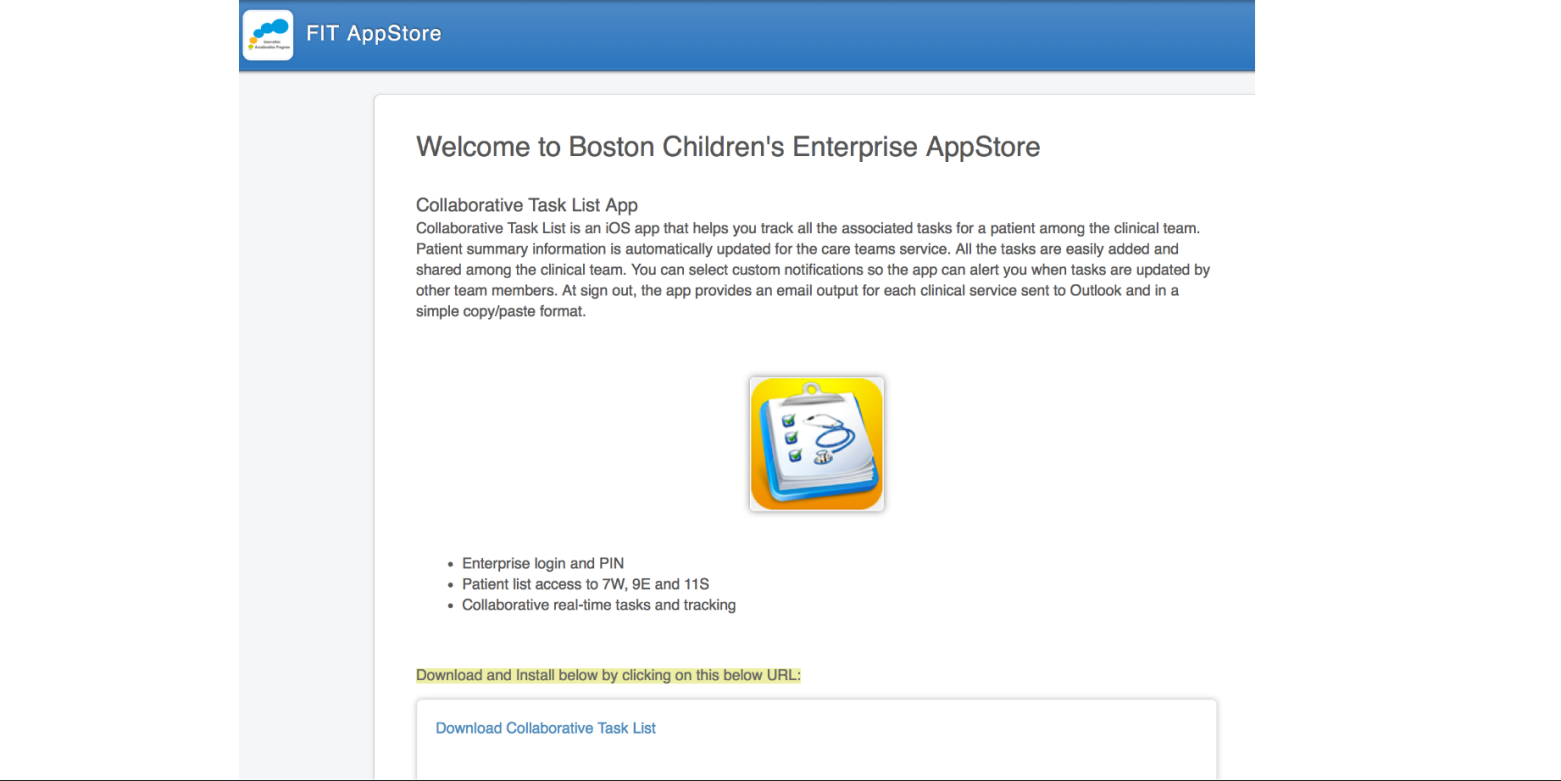
Boston Children's Hospital app store.

TaskList was developed using iOS that would typically imply the need to distribute the app via the iOS App Store. However, because the app was only for BCH employees, an internal OTA installation and update system was developed (Figure 6). It reduced the burden on both the users and IT.

##### Implement Remote Wipe Out Functionality

Because TaskList does not store any data locally on a device and uses no-cache, the functionality that is able to remotely wipe out the device was not required. In the future, when many departments join the pilot, the data get bigger and more time is needed to load the data from server. Consequently, storing some of the data locally on the device and using on-memory cache and implementing remote wipe out functionality will be mandatory.

##### Implement Ability to Disconnect and Block a User Anytime

In the TaskList project, as residents leave the organization or no longer have access privileges to the app, their credential must be deleted. We built this functionality by creating a dynamic user table so that an administrator is able to manage user access status.

## Discussion

### Pricipal Findings

The benefits of using mobile devices in a hospital continue to be recognized as a growing necessity for the future of health care delivery. A recent study [30] found that 60% of physicians reported avoiding at least one adverse drug error per week by using information found on mobile apps. In the same survey, physicians also reported saving time by using smartphone medical apps, with one in two stating they saved 20 minutes or more daily. For a busy primary care physician, that could mean the ability to see two to four more patients each day. Still, the widespread use of mobile apps and devices that are fully integrated into a comprehensive hospital information system or EMR remains a work in progress.

As a response to the aforementioned situation, we developed the guideline to build an app that complies with BYOD concerns and policies in a health care organization. The guideline helps both developers and security administrators balance between maximizing the use of personal devices in hospital settings and minimizing the security risk of BYOD with PHI. Using the guideline, we successfully implemented a mobile, collaborative, and real-time app called TaskList and piloted the app in a busy inpatient ward in a pediatric hospital. During the development and deployment processes, we also gained valuable knowledge and experience to build future apps that require similar robust security measures. Even though this manuscript focuses on custom developed apps for BYODs, this guideline is also relevant for vendors wanting to deploy any apps running on hospital supplied devices or BYODs.

Finally, through the application of the guideline to developing TaskList, we learned that there is no single solution that will solve all the BYOD issues in health care organizations, but a combination of legal policy, proper administrative procedure supported by advance technologies such as MDM apps, education, advance security detection, and ensuring all apps comply with established BYOD guidelines can help mitigate multiple security concerns.

### Conclusions

This was our first initiative and is a preliminary approach to implement BYOD in BCH; thus, we will continue to investigate and analyze how to best integrate personal devices into the BCH environment. This paper demonstrates one example given the BYOD technologies at BCH in 2014 and we are aware that as the technologies advance so will the custom app development approach with BYOD. We expect that as the BYOD needs continue to grow within health care organizations, more apps will adhere to security standards that protect medical information. Until there are industry-accepted guidelines we will use this BCH BYOD guideline to inform our enterprise mobile development design approach. At the same time, we will keep it updated to ensure the guideline meets the latest technology and research standards.

## Abbreviations

AES: advanced encryption standard
APNS: Apple push notification system
BA: business associate
BAA: business associate agreement
BCH: Boston Children’s Hospital
BYOD: bring-your-own-device
EMR: electronic medical record
GCM: Google Cloud Messaging
HIPAA: Health Insurance Portability and Accountability Act
HITECH: Health Information Technology for Clinical Health Act
iOS: Apple operating system
ISD: information services department
IT: information technology
LAN: local area network
MDM: mobile device management
OTA: over-the-air
PHI: protected health information
PIN: personal identification number
RBAC: role-based access control
SSL: secure sockets layer
VPN: virtual private networks
